# Fish Skin Grafts Affect Adenosine and Methionine Metabolism during Burn Wound Healing

**DOI:** 10.3390/antiox12122076

**Published:** 2023-12-05

**Authors:** Aristotelis Kotronoulas, Adrián López García de Lomana, Helga Kristín Einarsdóttir, Hilmar Kjartansson, Randolph Stone, Óttar Rolfsson

**Affiliations:** 1 Center for Systems Biology, Medical Department, University of Iceland, Sturlugata 8, 102 Reykjavik, Iceland; 2 Kerecis^®^, 101 Reykjavik, Iceland; 3 US Army Institute of Surgical Research, JBSA Fort Sam Houston, TX 78234, USA

**Keywords:** wound healing, burn wounds, metabolomics, adenosine and methionine salvage pathways, untargeted metabolomics

## Abstract

Burn wound healing is a complex process orchestrated through successive biochemical events that span from weeks to months depending on the depth of the wound. Here, we report an untargeted metabolomics discovery approach to capture metabolic changes during the healing of deep partial-thickness (DPT) and full-thickness (FT) burn wounds in a porcine burn wound model. The metabolic changes during healing could be described with six and seven distinct metabolic trajectories for DPT and FT wounds, respectively. Arginine and histidine metabolism were the most affected metabolic pathways during healing, irrespective of burn depth. Metabolic proxies for oxidative stress were different in the wound types, reaching maximum levels at day 14 in DPT burns but at day 7 in FT burns. We examined how acellular fish skin graft (AFSG) influences the wound metabolome compared to other standard-or-care burn wound treatments. We identified changes in metabolites within the methionine salvage pathway, specifically in DPT burn wounds that is novel to the understanding of the wound healing process. Furthermore, we found that AFSGs boost glutamate and adenosine in wounds that is of relevance given the importance of purinergic signaling in regulating oxidative stress and wound healing. Collectively, these results serve to define biomarkers of burn wound healing. These results conclusively contribute to the understanding of the multifactorial mechanism of the action of AFSG that has traditionally been attributed to its structural properties and omega-3 fatty acid content.

## 1. Introduction

Acute thermal injuries requiring medical care are a leading cause of morbidity worldwide, with an estimated 180,000 deaths every year [1]. In the last few decades, the survival rate of patients has increased due to significant innovations in burn wound care and treatment. An important contribution to better quality wound care has been the advancements in biomaterials used to improve burn wound healing processes [2,3,4]. Xenografts of different animal origins, such as acellular fish skin graft (AFSG) or fetal bovine dermis (FBD), are among those used due to their beneficial healing properties for various types of burn wounds [5,6].

Burn wounds are classified as superficial, superficial partial thickness, deep partial thickness (DPT), or full thickness (FT). Superficial or epidermal burns involve only the epidermal layer of skin. They do not blister but are painful, dry, pink to red, and blanch with pressure. Superficial partial-thickness burns characteristically form blisters within 24 h between the epidermis and dermis; they are painful, red, weeping, and blanching with pressure. DPT extends into the deeper dermis and damages hair follicles and glandular tissue. FT burns extend through and destroy all layers of the dermis and often injure the underlying subcutaneous tissue [7]. Accordingly, the need for surgical grafting and healing of the wound is largely determined by the depth of the burn injury [8,9].

During burn wound healing, patients with larger burns may enter a prolonged period of hypermetabolism, chronic inflammation, or lean body mass wasting, all of which may impair the process [10] through delayed re-epithelialization [6,11]. Additionally, an increased susceptibility to infection due to altered immune status may lead to sepsis or further systemic inflammation [12], with a significantly high risk of mortality [13,14]. The ability to accurately monitor the quality of burn wound healing over time can lead to therapeutic interventions that minimize scar tissue formation and predict whether a burn wound will spontaneously heal, resulting in improved individual patient care [15].

Measuring biomarkers in burns may help to understand how the wound healing process occurs and indicate the pathophysiology of organ dysfunction development [16,17,18]. Numerous studies have evaluated specific biomarkers and their relationships to clinical variables (e.g., burn severity, stress, and sepsis) and survival through comparative analysis of patients vs. controls and during burn wound healing. These biomarkers include cytokines, growth factors, structural proteins, and hormones that have primarily been investigated in blood [19,20,21,22] and, to a lesser extent, in urine [23,24], blister fluid [25], and saliva [26]. The altered metabolism of cells, along with metabolic changes to the wound microenvironment, is required to ensure timely nutrient supply and waste disposal [27]. Biomaterials such as AFSGs and FBD are broken down and integrated during wound healing and thus contribute to an altered nutrient supply, reactive oxygen species (ROS) homeostasis, and the wound microenvironment. With respect to nutrients, in particular, biomaterials can be viewed as a platform to introduce metabolic interventions that boost skin regeneration that may, in part, occur through modulation of oxidative stress [28]. We have shown that an AFSG, rich in omega-3 polyunsaturated fatty acids, leads to altered lipid temporal profiles during healing [29]. However, AFSGs and FBD contain multiple other nutrients, such as amino acids, that are likely to affect wound healing differentially.

Developments in omics techniques have collectively led to the discovery of biomarkers in the fields of biomedicine and preventive medicine [30]. Few investigations have, however, been performed to assess metabolomic changes during the healing of burn wounds directly from skin biopsies. Research has mainly been focused on identifying changes on the systemic level and principally in blood. In 2010, Liu et al. [31] used high-resolution TOF mass spectrometry and untargeted metabolomics to identify nine characteristic metabolites in rat plasma that could distinguish between burned septic and non-septic groups. Moreover, an untargeted lipidomic methodology implemented by Qi et al. [32] revealed that unsaturated and free fatty acids in sera of specific cohorts of burn patients were associated with increased risk for chronic hypermetabolism and poor healing outcomes. To the best of our knowledge, however, the metabolomic analysis of burn wound healing has not been studied on wound tissue directly.

Here, we applied an untargeted metabolomics discovery approach to study the metabolic changes that occur during wound healing directly from biopsies collected from DPT and FT porcine burn wounds over a period of 60 days of healing. The porcine burn model and associated healing phenotypes have recently been described separately [29,33]. We performed a comparative statistical analysis of the metabolomics data, consisting of detected mass-to-charge ratios (i.e., *m*/*z* features), to identify *m*/*z* features that are altered during the course of healing. We subsequently annotated the *m*/*z* features to identify biomarkers of the wound healing process irrespective of treatment and identify metabolic alterations due to the use of particular xenografts.

## 2. Materials and Methods

### 2.1. Experimental Design

The experimental design and execution have been previously described [29,33]. The animal experiment was previously performed under a US Army Institute for Surgical Research (USAISR) approved IACUC protocol (A-16-021-TS5 approved 5 May 2017). Research was conducted in compliance with the Animal Welfare Act, the implementing Animal Welfare Regulations, and the principles of the Guide for the Care and Use of Laboratory Animals. The Institutional Animal Care and Use Committee approved all research conducted in this study. The facility where this research was conducted is fully accredited by AAALAC International. Animals were housed individually in a temperature-controlled environment with a 12 h light/dark cycle in the AAALAC-approved vivarium at the USAISR, with access to water and food ad libitum.

Before burning, chemical depilation (Nair™) was used to remove hair from the dorsum of the animals, and the skin was rinsed with sterile water. Four DPT and six FT burn wounds (5 cm × 5 cm) were created on the dorsum of anesthetized Yorkshire pigs (N = 6) using appropriate pain control methods. The burn depth and treatments were randomized on the back of the pig. Burns were then debrided twenty-four hours post-burn. DPT burn wounds were treated with either omega-3-rich AFSGs or FBD (two wounds for each pig, respectively). After 7 days, AFSGs were reapplied, and all wounds were allowed to heal by secondary intentions. The FBD xenografts used were commercially available under the trademark Primatrix (TEI Biosciences Inc, Boston, MA, USA). FT burn wounds received different treatments. FT burn wounds were treated with either AFSGs or cadaver skin (two wounds for each pig, respectively) until Day 7, and then we grafted the wounds with a 1.5:1 meshed split-thickness skin graft. The remaining 2 FT wounds were grafted with a 3:1 meshed split-thickness skin graft with an additional piece of AFSG covering the graft in a sandwich fashion. We acquired cadaver skin by harvesting skin grafts with a pneumatic dermatome from recently euthanized Yorkshire pigs. All experimental manipulations were performed under anesthesia, with pain control as needed. Finally, punch biopsies were collected from each wound on Days 7, 14, 21, 28, and 60 and immediately frozen at −80 °C.

### 2.2. Sample Preparation and UPLC-MS/MS Analysis

Punch biopsies were thawed at room temperature, weighed, and subsequently homogenized using plastic pestles in 0.5 mL of 70% MeOH for 1 min. We set the supernatant aside after centrifugation at 13,000 rpm for 10 min. We repeated the homogenization process on the pellet with another 0.5 mL of 70% MeOH, and both supernatants were combined, dried under vacuum, and immediately frozen at −80 °C until further analysis.

Samples were resuspended in 200 μL of 50:50 H_2_O:ACN and analyzed in random order using an Acquity ultra-high-performance liquid chromatography (UPLC) system coupled to a Synapt G2 quadrupole time-of-flight mass spectrometer (Waters, Manchester, UK) with an electrospray ionization interface (ESI). Chromatographic separation was achieved on hydrophilic interaction liquid chromatography (HILIC) using an Acquity amide column, 1.7 μm (2.1 × 150 mm; Waters), using as mobile phase A 100% ACN and B 100% H_2_O, both containing 0.1% formic acid. We used the following elution gradient: 0 min 99% A, 7 min 30% A, 7.1 min 99% A, and 10 min 99% A. The flow rate was 0.4 mL/min, the column temperature was 45 °C, and the injection volume was 7.5 μL. All samples were analyzed in both positive and negative modes. The mass spectrometer operated using a capillary voltage of 1.5 kV, and the sampling cone and the extraction cone were 30 and 5 V, respectively. The cone and the desolvation gas flow were 50 and 800 L/h, respectively, while the source and desolvation gas temperatures were 120 and 500 °C, respectively. MS spectra were acquired in centroid mode from *m*/*z* 50 to 1000 using a scan time of 0.3 s. Leucine enkephalin (2 ng/μL) was used as lock mass (*m*/*z* 556.2771 and 554.2615 in positive and negative modes, respectively). Argon served as a collision gas in both positive and negative modes. For the high-definition data-independent acquisition (DDA) experiments, the collision energy in the trap cell was off, and in the transfer cell, it ranged from 10 to 30 eV for the positive mode and from 25 to 40 eV for the negative mode.

### 2.3. Data Processing and Statistical Analysis

The obtained chromatograms were aligned, and *m*/*z* features were extracted with the program XCMS [34]. Quality control (QC) samples, consisting of pooled metabolite extracts from all samples, were injected every seven samples to evaluate the data integrity and allow batch normalization. All *m*/*z* features that were found in less than 80% of QC samples and those with a relative standard deviation (RSD) < 25% among the QC samples were removed. Normalization of the acquired intensities was performed based on LOESS methodology for metabolomics, included in the NormalizedMets package for R [35]. (Supplementary Table S1).

For statistical analysis, we used the program Metaboanalyst v5.0 [36]. To identify metabolic changes during wound healing, we applied a 2-way ANOVA in order to account for the changes due to the variables of “Time” (5 different time points) and “Treatment” (two different treatments for each wound type). Student’s *t*-tests were performed to identify metabolic features that changed in abundance at different timepoints. On the volcano plot visualizations, we highlighted responding features as those that showed significance (Student’s *t*-test; Bonferroni correction, *p* < 0.1) and an absolute fold-change (FC) greater than 1.5.

Metabolic pathway analysis was performed on the MetaboAnalyst web page (version 5.0) [36]. The compound name selection was carried out with exact matching, and the *Homo sapiens* (human) library was used. Overrepresentation analysis was determined using a hypergeometric test, and the topological analysis was carried out using relative betweenness centrality.

In order to identify temporal trajectories, we computed the z-score of each metabolite abundance across the studied samples. We identified metabolite clusters using hierarchical clustering using the seaborn. Clustermap function and complete and cosine are options for the linkage method and distance metric, respectively. We identified the optimal clustering partition using the consensus across three metrics: Silhouette score, Calinski–Harabasz index, and Davies–Bouldin index. Code to reproduce metabolite trajectory inference is available in the GitHub repository https://github.com/adelomana/healing (accessed on 28 November 2023).

### 2.4. Feature Annotation/Identification of Metabolites

Detected *m*/*z* features were annotated using our previously published in-house database [37]. For *m*/*z* features not covered within that database, we used xMSAnnotator [38], an R-based package that utilizes the intensities from the isotopic patterns and their possible coeluting adducts in order to annotate each feature putatively. Proposed structures with error mass lower than 10 ppm were then selected (level 3 annotation). To improve annotation confidence of the statistically significant *m*/*z* features annotated with xMSAnnotator, we then followed a combination of two strategies: (1) We employed the CAMERA algorithm [39] for to the output of the XCMS peak identification process, and peaks were then grouped together based on the PC group number. All masses within a PC group were then introduced as a fragment list into the Metlin v 2.0 precursor search engine. (2) We examined the fragmentation pattern acquired from DDA analysis of the sample pools. The list of *m*/*z* fragments corresponding to each feature was compared to the Metlin online database and in-house library. Moreover, the DDA files were uploaded to GNPS servers for automatic identification. Within GNPS, a minimum of 5 common fragment masses between the uploaded experimental spectra and one of the GNPS libraries were accepted. The combination of strategies 1 and 2 led to annotation levels 1 and 2. The methodology and the respective levels of annotation were followed as in Schymanski et al. [40].

## 3. Results

### 3.1. Amino Acid Metabolism Is Altered during Healing Irrespective of Burn Wound Depth

The untargeted metabolomics analysis identified 1367 *m*/*z* features (1008 and 359 features in positive and negative ionization mode, respectively) across all analyzed biopsies. We first focused our analysis on the wound type. A two-way analysis of variance (ANOVA) for time and treatment as influences identified 318 *m*/*z* features significantly associated with time in DPT and 248 in FT burn wounds (two-way ANOVA; Benjamini-Hochberg corrected (Q = 0.05); *p* < 0.05; Supplementary Table S1). Only a single *m*/*z* feature was significantly associated with treatment influence in DPT across all sampled time points. These features are referred to as *features of interest* for either the DPT or the FT burn wounds in the subsequent sections.

Next, we set out to annotate the *m*/*z* features of interest associated with time (see Section 2.4). In the DPT wounds, 100 features of interest could be annotated at a confidence level of 3. These compounds fall into diverse metabolite classes, including amino acids, peptide analogs, lipids, lipid-like molecules (including fatty acyls and steroids), as well as carboxylic acids and derivatives. However, a total set of 26 features of interest were annotated at the higher confidence levels of 1 or 2, based upon MSMS spectral matches and/or in-house database matches (Table 1). Similarly, 29 features of interest could be identified with high confidence in the FT wounds (Table 2), while approximately 109 had a lower confidence level of 3. These fall into similar metabolite classes as those identified in the DPT wounds. A list of all annotated features, including those with lower annotation scores, is supplied in Supplementary Table S2.

**Figure 1:**
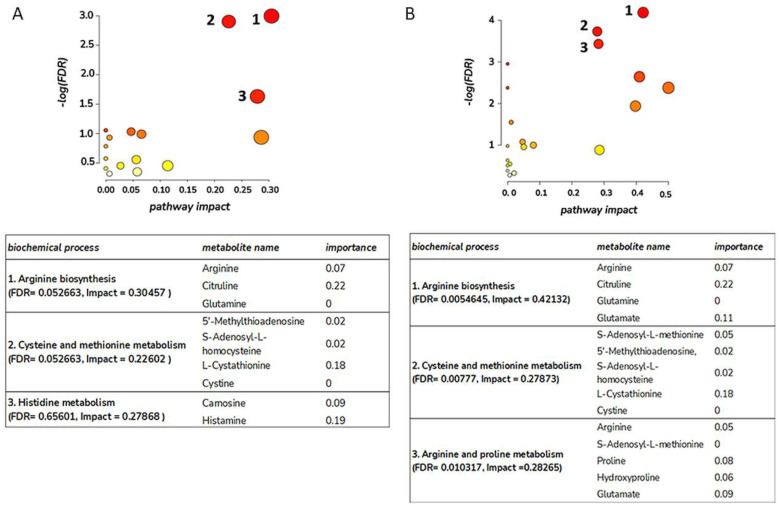
Metabolic pathway analysis for the annotated features of interest in deep partial-thickness burn wounds (**A**) and full-thickness burn wounds (**B**). Pathway impact amalgamates centrality and pathway enrichment outcomes, with elevated impact values signifying the pathway’s relative significance. The circle’s size signifies the pathway’s impact, while the color reflects its statistical significance, with red indicating increased significance. The analysis was created using MetaboAnalyst 4.0. The same metabolic pathways are impacted during wound healing, irrespective of wound type.

Moving forward with our analysis with features of interest and high confidence annotations (Table 1 and Table 2), we performed pathway analysis of metabolites identified during healing of each wound type (Figure 1). Arginine biosynthesis, followed by cysteine and methionine metabolism, was highlighted in both wound types. Arginine, citrulline, glutamine, and glutamate fall within the arginine biosynthesis pathway, while changes to S-adenosyl-L-methionine, 5′-methylthioadenosine, S-adenosyl-L-homocysteine, L-cystathionine, and L-cystine implied changes to cysteine and methionine metabolism. In the set of changing metabolites in FT burn wounds, the arginine and proline metabolism pathway was also enriched on account of arginine, proline, and hydroxyproline. Considering the overlap of identified metabolites in DPT and FT wounds and similar metabolic pathway enrichments, arginine, and proline metabolism are altered during burn healing, irrespective of wound type.

**Table 1:** Identified metabolites that changed in concentration during wound healing in deep partial-thickness burn wounds (Two-way ANOVA; Bonferroni corrected *p*-value < 0.05 for time variable). The temporal pattern of each metabolite in relation to Figure 2 is shown in the last column.

Metabolite	Kegg ID	Mode	*m*/*z*	Retention Time	*p*-Value	Temporal Pattern
Allantoin	C02350	neg	157.0367	3.4	2.0 × 10^−3^	U1
Arginine	C00062	pos	175.1193	5.0	3.0 × 10^−8^	U2
Histamine	C00388	pos	95.0610	4.7	1.0 × 10^−4^	U2
3,4-Dihydroxymandelic acid	C05580	pos	84.9606	5.8	8.0 × 10^−3^	P2
5′-Methylthioadenosine	C00170	pos	298.1026	4.8	2.0 × 10^−7^	P2
Valine/Betain	C00183	pos	118.0866	3.8	8.0 × 10^−11^	P2
Carnosine	C00386	pos	151.1390	5.0	4.0 × 10^−4^	P3
Citrulline	C00327	pos	150.0772	4.6	7.0 × 10^−3^	P2
Cystathionine	C02291	pos	223.0759	5.4	1.0 × 10^−5^	P1
Cystine	C00491	pos	151.9845	5.4	8.0 × 10^−5^	P2
Cytidine	C00475	neg	242.0792	4.7	6.0 × 10^−4^	P3
Glutamine	C00064	pos	148.0799	4.8	6.0 × 10^−3^	P2
Glycerophosphocholine	C12181	pos	258.1105	4.7	8.0 × 10^−3^	P1
Guanosine	C00387	pos	328.0643	3.9	3.0 × 10^−3^	P2
Hypotaurine	C00519	neg	108.0121	4.5	9.0 × 10^−10^	P2
Glycogen/Maltotetraose	C02052	neg	665.2152	5.6	5.0 × 10^−4^	P2
Maltotriose	C01835	neg	549.1679	5.3	1.0 × 10^−3^	P2
Oxidized glutathione	C00051	pos	556.1392	5.4	3.0 × 10^−3^	P2
PI(18:2/20:3)	#N/A	pos	887.5654	3.1	6.0 × 10^−3^	P3
pyrophosphate	C00013	neg	176.9356	4.4	7.0 × 10^−3^	P3
S-Adenosylhomocysteine	C00021	pos	238.0463	4.8	7.0 × 10^−7^	P3
LPC(18:2)	#N/A	pos	564.2877	5.1	9.0 × 10^−9^	D1
LPC(20:4)	#N/A	pos	588.6345	5.0	7.0 × 10^−12^	D1
Methyladenosine	C02494	pos	282.1220	3.4	4.0 × 10^−4^	D1

**Table 2:** Identified metabolites that changed in concentration during wound healing in full-thickness burn wounds (Two-way ANOVA; Bonferroni corrected *p*-value < 0.05 for time variable). The temporal pattern of each metabolite in relation to Figure 3 is shown in the last column.

Metabolite	Kegg ID	Mode	*m*/*z*	Retention Time	*p*-Value	Temporal Pattern
Histamine	C00388	pos	95.06082	5.1	8.80 × 10^−3^	U1
Serine	C00065	neg	104.03505	4.6	6.62 × 10^−5^	U1
Hypotaurine	C00519	pos/neg	110.02764/108.01206	4.4	1.60 × 10^−3^	U1
Histidine	C00135	pos/neg	156.07728/154.06188	5.0	2.42 × 10^−6^	U1
Allantoin	C02350	neg	157.03668	3.4	1.90 × 10^−3^	U1
Citrulline	C00327	neg	174.08813	4.6	3.48 × 10^−4^	U1
Arginine	C00062	pos	175.11927	5.0	8.57 × 10^−10^	U1
Cystathionine	C02291	neg	221.06041	5.4	2.19 × 10^−8^	P1
Cytidine	C00475	neg	240.92911	4.0	9.00 × 10^−3^	P1
Hydroxyproline	C01157	neg	130.05016	4.4	1.17 × 10^−5^	P3
S-adenosyl methionine	C00019	pos	708.63496	NA	5.15 × 10^−6^	D1
Glutamine	C00064	pos	148.0799	4.8	3.25 × 10^−5^	D2
Pantothenic acid	C00864	pos	220.11887	2.5	5.20 × 10^−3^	D2
5′-Deoxy-5′-methylthioadenosine	C00170	pos	298.10262	5.3	4.93 × 10^−12^	D2
Oxidized glutathione	C00051	pos	777.8926	NA	4.83 × 10^−4^	D2
Leukotriene C4	C02166	pos	828.9304	NA	8.51 × 10^−6^	D2
Valine/Betaine	C00183	pos	118.08662	3.8	1.92 × 10^−11^	D3
Glutamate	C00025	pos/neg	148.06088/146.04521	4.4	3.20 × 10^−7^	D3
Cystine	C00491	pos	151.98453	NA	3.12 × 10^−3^	D3
3,4-Dihydroxymandelic acid	C05580	pos	125.98727	5.8	5.15 × 10^−6^	D3
Guanine	C00242	pos	152.05761	5.4	2.44 × 10^−4^	D3
S-Adenosylhomocysteine	C00021	pos	238.04632	4.8	2.29 × 10^−4^	D3
N-acetyl aspartate	C01042	neg	359.99008	4.2	4.96 × 10^−4^	D3
CMP	C00055	pos/neg	324.06018/322.04423	5.3	5.00 × 10^−3^	D3
NADH	C00004	pos	362.0896	5.2	3.00 × 10^−3^	D3
GMP	C00144	pos	364.06663	5.3	6.00 × 10^−4^	D3
Proline	C00148	neg	385.93804	NA	3.00 × 10^−3^	D3

### 3.2. Metabolite Trajectories of Wound Healing Are Different across DPT and FT Burn Wounds

**Figure 2:**
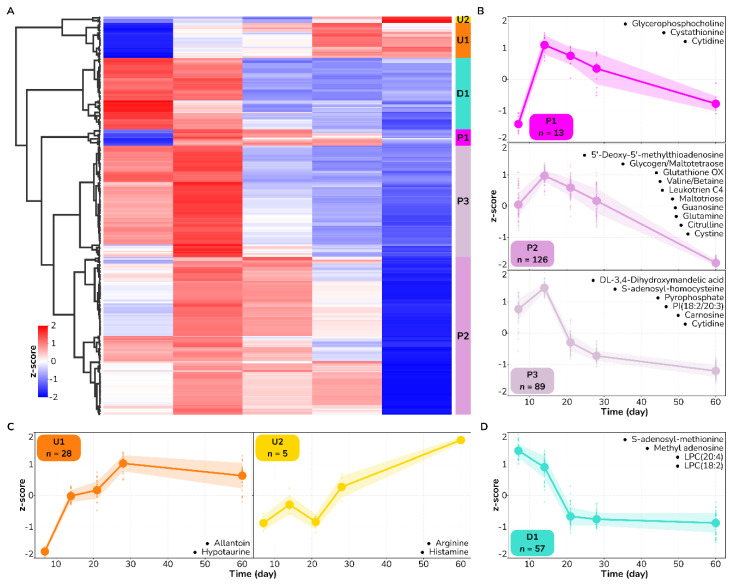
Temporal patterns of metabolic changes across the wound healing process in partial -thickness burn wounds. (**A**) Heatmap showing the relative change (z-score) of the 318 statistically significant time-changing metabolic features (columns). Agglomerative clustering on features identified six typical trajectories in DPT burn wounds. (**B**–**D**) Each cluster representative of a unique trajectory is labeled with a letter (U, upregulated; D, downregulated; and P, peak) and a number indicative of how early metabolites respond to the healing process. Annotated metabolites from Table 1 are mapped to each trajectory and highlighted in their corresponding subpanels.

**Figure 3:**
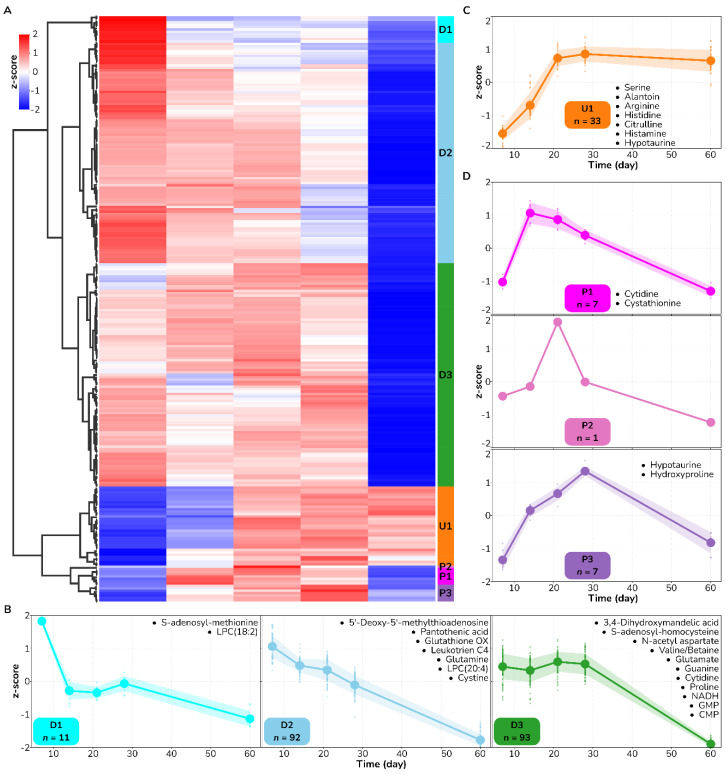
Temporal patterns of metabolic changes across the wound healing process in full-thickness burn wounds. (**A**) A heatmap showing the relative change (z-score) of the 248 statistically significant time-changing metabolic features (columns). Agglomerative clustering on features identified seven typical trajectories in FT burn wounds. (**B**–**D**) Each cluster representative of a unique trajectory is labeled with a letter (U, upregulated; D, downregulated; and P, peak) and a number indicative of how early metabolites respond to the healing process. Annotated metabolites from Table 2 are mapped to each trajectory and highlighted in their corresponding subpanels.

To identify temporal patterns of metabolic change across the wound healing process, we performed cluster analysis of the wound healing features of interest in both the DPT and FT datasets. Specifically, we combined agglomerative clustering coupled with cluster partition evaluation using three different clustering goodness criteria—Silhouette score, Calinski and Harabasz score, and Davies–Bouldin index (see Section 2 for details)—to determine the different metabolic trajectories. Consistently across both types of injury, we found three major patterns of metabolic change across the wound healing process: (i) An upregulated monotonic saturating pattern (labeled as U); (ii) A downregulated monotonic saturating pattern (labeled as D); (iii) A non-monotonic pattern peaking at a particular time during the healing process (labeled as P) (Figure 2 and Figure 3). Further, each of these major patterns could be split into up to three trajectories, depending on the timing of the response.

In DPT wounds, most metabolic features (228 out of 318, 72%) displayed a non-monotonic trend, exhibiting a maximum value on Day 14 of the healing process (Figure 2). Within this pattern, we identified three different trajectories that were separated depending on their behavior on Day 7—either above (P1), near (P2), or below average value (P3). Metabolites involved in regulating oxidative stress included glutathione, carnosine, cystathionine, and cystine, which all decreased following day 14 of healing. All the metabolites that mapped to the biochemical processes identified through the pathway analysis (Figure 1), apart from arginine and histamine, displayed these non-monotonic patterns indicative of flux alterations through respective pathways during healing. Other metabolic temporal patterns identified in DPT wounds, with 33 metabolic features, displayed upregulated trends. In this case, we distinguished an early and late response with representative metabolites allantoin and hypotaurine in the early response (U1 trajectory, average values observed by Day 21) and arginine and histamine in the late response trajectory (U2 trajectory, average values observed by Day 28). Lastly, we identified a downregulated trajectory (D1) with 57 metabolic features, including lysophosphatidyl cholines and S-adenosyl-methionine. Altogether, these results identify metabolic biomarkers of DPT burn wound healing and suggest that oxidative stress reaches a maximum at day 14 of healing.

Next, we applied the same quantitative analysis to the metabolic dataset derived from the FT wound biopsies (Figure 3). Most of the metabolic features (196 out of 248, 79%) followed a downregulated saturating trend, contrary to the non-monotonic pattern observed in the DPT wounds. We found three characteristically different trajectories within this pattern based on how early they transitioned from high to low levels. Of the metabolites that mapped to enriched biochemical processes from Figure 1, S-adenosyl methionine falls into the D1 trajectory, which was the earliest to respond within this pattern with values below average already in Day 14. Glutamine, 5-MTA, and cystine fall into the next responding trajectory (D2), whose metabolite levels reached below average by Day 28. Lastly, glutamate, SAH, and proline had late-responding metabolite trajectories (D3), whose average values were still above average on Day 28. Alongside downward trajectories, we distinguished an upward trajectory (U1) containing 33 metabolic features that consistently increased in the form of a sigmoidal shape during the wound-healing process. We identified arginine, citrulline, and histamine as representative metabolites in this trajectory. Finally, we identified a smaller set of 19 metabolites that followed three distinct non-monotonic dynamical patterns depending on how early metabolites reached their maximum values. The earliest trajectory, P1, containing cytidine and cystathionine, peaked on Day 7, while the P3 trajectory, containing hydroxyproline, reached its maximum value on Day 28. We conclusively uncovered here a set of distinct representative metabolic temporal patterns in both DPT and FT burn wounds, in addition to various metabolites that can be used as biomarkers of the healing process.

### 3.3. Methionine Salvage Is Affected by Burn Wound Treatment on Day 7

While the two-way ANOVA implied few changes to metabolic profiles on account of treatment, our previous analysis of lipid mediators had demonstrated changes on account of the treatment at Day 7. We, therefore, investigated treatment-specific differences in metabolic profiles using a different approach by comparing metabolite intensities in the differentially treated wounds at each time point using a Student’s *t*-test and visualizing the results using a volcano plot (Supplementary Table S2 and Supplementary Figure S1). These analyses identified 585 and 315 *m*/*z* features that were significantly different on account of treatment in the biopsies collected on Day 7 from the DPT and FT wounds, respectively. Notably, a similar comparison for the samples collected on the following days of wound healing did not reveal any differences between detected *m*/*z* features, which reconciles with the results we observed taking the two-way ANOVA approach. Furthermore, this pattern is biologically consistent with other studies that showed that changes to lipid mediators take place on Day 7 of burn wound healing [29].

**Figure 4:**
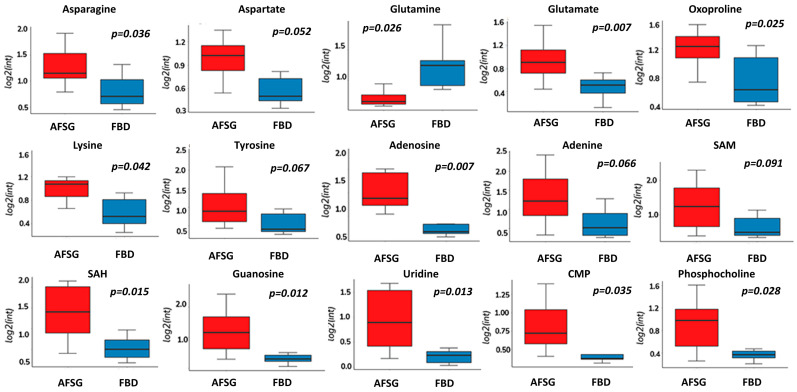
Metabolites showing differences in abundance in DPT burn wounds on Day 7 when treated with AFSG compared to FBD. Red is AFSG, and blue is FBD. Fold change > 1.5, Students’ *t*-test, FDR Bonferroni, *p* < 0.1.

**Figure 5:**
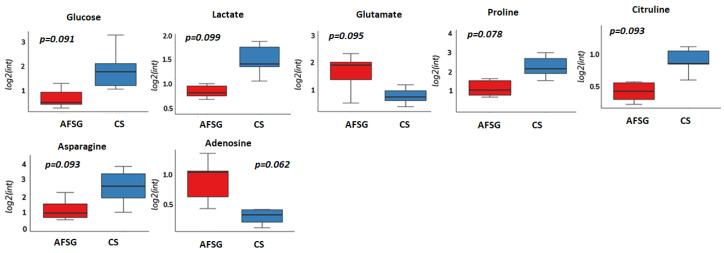
Metabolites showing differences in abundance in full-thickness burn wounds on Day 7 when treated with AFSG compared to CS. Red is AFSG, and blue is CS. Fold change > 1.5, Students’ *t*-test, FDR Bonferroni, *p* < 0.1.

Of the identified treatment-responding metabolic features of interest, fifteen and seven features were annotated with confidence levels 1 or 2 in the DPT and FT wounds, respectively. In the DPT wounds, the identified metabolites consisted of amino acids, nucleotide derivatives, and phosphocholine and were all increased in the AFSG-treated wounds apart from glutamine (Figure 4). In the FT wounds, adenosine and glutamate were higher on Day 7 in the ASFG-treated wounds, while glucose, lactate, proline, citrulline, and asparagine were lower compared to cadaver skin-treated wounds (Figure 5).

**Figure 6:**
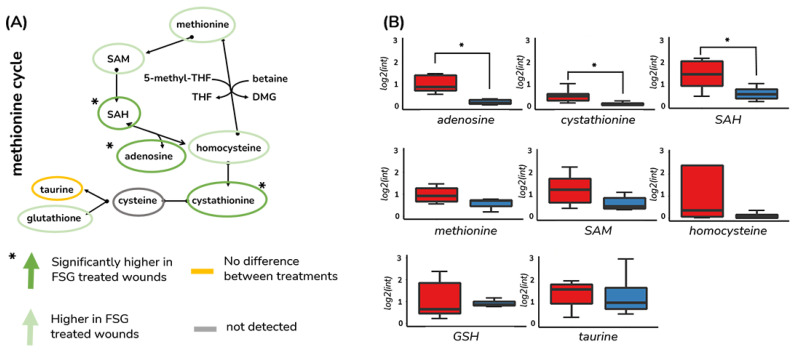
Metabolites within the methionine salvage pathway increase in DPT burn wounds treated with AFSG compared to FBD. (**A**) Schematic representation of the methionine salvage pathway and the observed differences in AFSG compared to FBD treatment in DPT wounds on Day 7. (**B**) Abundance differences of identified compounds within the pathway in DPT wounds on Day 7 (* Students’ *t*-test, FDR Bonferroni, *p* < 0.05). Red is AFSG, and blue is FBD. The calculated normalized intensities for taurine did not show differences among the wounds, while cysteine was not detected.

In the DPT burn wounds, we noted that four of the metabolites identified take part in methionine metabolism, which was also found to be enriched during healing (Figure 1). More specifically, adenosine, s-adenosyl homocysteine, S-adenosyl methionine, and cystathionine take part in the methionine salvage pathway. We, therefore, expanded our analysis of metabolites in this pathway and found that a total of eight metabolites in this pathway could be annotated with high confidence. Seven of these were increased in the AFSG treated DPT wounds, suggesting that the treatment of DPT burn wounds with AFSG affects methionine salvage (Figure 6).

## 4. Discussion

Here, we took an untargeted LC-MS discovery approach to capture metabolic changes in burn wound healing across a 60-day period. A subset of detected *m*/*z* features changed in intensity during healing, of which roughly 5–10% could be confidently annotated. Irrespective of the wound type, several of the identified metabolites, belonging to metabolic pathways and involving in arginine metabolism and metabolism of the sulfur-containing amino acids cysteine, methionine, and hypotaurine, are indicative of changes to reactions that modulate reactive nitrogen and oxygen species during healing. Our data showed that metabolic changes during healing can be described with six and seven distinct metabolic trajectories for DPT and FT burn wounds, respectively. These trajectories contained hundreds of metabolic features, which we identified mainly as amino acids, organic acids, and nucleotide derivatives (Supplementary Table S1). In addition, we explored how the wound metabolome is affected by the different treatments that were used to support wound healing. For both types of wounds, treatment affected the concentration of selected amino acids, nucleotides, and nutrients within wounds on Day 7, while later stages of healing were unaffected by the type of graft.

Recently, our group published an evaluation of the healing process of the same wounds, following their progress with histological approaches [33]. Changes were observed in DPT wound closure rates and epithelialization that occurred faster in AFSG-treated wounds on Days 14 and 21, respectively. Nevertheless, complete wound closure was observed after 60 days for all treatments and wound types [33]. Changes to lipid mediator concentrations in these same wounds showed that treatment-specific changes were restricted to Day 7. Temporal changes to lipid mediators during healing, however, showed a maximum after 14 and 21 days for most lipid mediators measured independent of wound type and treatment [29]. Combined with and in light of a previous study on AFSG-treated wounds [41], these studies concluded that AFSG wound treatment results in faster macrophage subsidence and resolution of inflammation [42,43].

In the current study, we first focused our analysis on determining the metabolic profiles of wound healing in DPT and FT wounds, independent of treatment. The identified metabolites and their trajectories, as defined by hierarchical cluster analysis, reflect changes in biochemical processes that underlie wound healing and contribute to the different wound healing phases. Specifically, pathway enrichment analysis for both DPT and FT showed that the arginine and histidine metabolisms were two of the most affected processes during healing, both of which have been reported in the context of wound healing. Arginine metabolism coincides with macrophage infiltration/subsidence in wounds. Changes to arginine metabolism are primarily attributed to changes in the activities of arginase-1 (ARG1) and nitric oxide synthase (NOS), generating ornithine, citrulline, and nitric oxide (NO). We identified changes to both arginine and citrulline in DPT and FT wounds indicative of changes to NOS synthesis, while ornithine was not detected. In the DPT wounds, arginine increased steadily during healing (U2 pattern), while citrulline peaked at day 7 (P2 pattern). NO produced from arginine aids the transition of a wound from the acute inflammatory phase to the proliferative phase of wound healing [44,45] and is central to angiogenesis [46]. Given the trajectory of citrulline, our results suggest that this transition occurs following day 7 of DPT burn wounds. As opposed to DPT wounds, arginine and citrulline increased steadily until day 21 in FT wounds (U1 pattern), indicating different dynamics within this pathway during the healing of FT wounds. One reason for this could be the enhanced requirement for arginine contribution to proline for extracellular matrix synthesis in FT wounds.

Like NO, histamine is a well-described regulator of the immune response. In DPT burn wounds, histamine had a U1 pattern similar to arginine, while in FT burn wounds, histamine and its precursor, histidine, followed the U1 pattern, again similar to arginine metabolism in FT wounds. Histidine levels are associated with histamine formation, which is catalyzed by histidine decarboxylase expressed in mast cells and basophiles but is also synthesized and released more slowly by other resident cells of the skin, including keratinocytes. The profiles we observed for histamine may indicate a slow release in DPT burn wounds, while in FT wounds, histamine profiles showed a more drastic increase up until Day 21, possibly on account of the faster cell infiltration into the DPT compared to FT burn wounds.

Histidine is also a precursor for the dipeptide carnosine that reached a maximum at day 14 and then decreased in DPT wounds (P3 pattern). Carnosine is a natural antioxidant with anti-inflammatory properties that has been shown to reduce lipid peroxidation. Its temporal profile suggests that oxidative stress drops following day 14 in DPT burn wounds, consistent with the resolution of inflammation during healing. A similar profile was observed for oxidized glutathione (P3 pattern) that steadily decreased in PT burn wounds following Day 14 and Day 7 in FT burn wounds (D2 pattern). Similar patterns were observed for the glutathione precursors glutamine, cystine, and metabolites of the trans-sulphuration pathway. These patterns are in accordance with changes to reactive oxygen species that accompany the resolution of inflammation following injury due to enhanced macrophage and neutrophil activity and are attributed to myeloperoxidase activity [47].

Some of the identified metabolites showed quite drastic changes in concentration associated with re-epithelialization and wound healing. These metabolites serve as potential biomarkers for burn wound healing. In DPT burn wounds, these include metabolites that fall into the U1, D1, and P3 patterns, such as hypotaurine, S-adenosyl methionine, and carnosine, respectively. In FT wounds, these included metabolites that fall into the U1 and D1 patterns, such as arginine and S-adenosyl methionine. Currently, no biochemical markers of burn wound healing have been implemented in clinical practice. For this purpose, the identified metabolites here could be pursued further in future studies with the aim of quantitatively determining the change in concentrations in these metabolites during healing to determine accurate concentration ranges associated with healing for diagnostic monitoring.

Further, we investigated metabolic changes due to the treatment on Day 7, seeking to explain increased wound closure rates and enhanced re-epithelialization when burn wounds are treated with AFSG [33]. Comparative analysis of the metabolic profiles at each timepoint for both types of wounds revealed differences between treatments in the first week after the wounds were created, while no differences were reported for the rest of the healing process. This is in accordance with our previous lipid mediator study [29]. During the first phase of wound healing, multiple successive processes, including hemostasis, phagocytosis, inflammation, and efferocytosis, are initiated and terminated. Taken together, our data suggest that biomaterials-based treatments alter the metabolome in the early stages of healing and modulate the intrinsic and/or extrinsic metabolic environment to enhance healing.

Out of the metabolites that we were able to identify, it is noteworthy that adenosine was increased in both DPT and FT wounds where AFSG was used as treatment. Adenosine is a well-described mediator of the immune response [48,49] and a modulator of oxidative stress [50,51,52]. In elegant mechanistic studies at the turn of the century, Adenosine A_2A_ receptor-induced signaling was shown to enhance wound healing rates in excisional and diabetic wounds in rats [53] and suppress inflammation [54]. Knockdown of the A_2A_ receptor in mice was also shown to decrease excisional wound healing rates and microvessel formation following adenosine A_2A_ receptor antagonization compared to wild-type mice [55]. The anti-inflammatory role of adenosine receptors has since been reported in many cases. Adenosine attenuates ROS through A_2a_ receptor activation [56]. In macrophages, their activation blocks the release of proinflammatory mediators, including TNF-, IL-6, and IL-12, with simultaneous release of the anti-inflammatory cytokine IL-10 [45,57]. Adenosine receptor activation has also been shown to reduce neutrophil adhesion, activation, and infiltration [58,59,60]. In burn wounds specifically, Adenosine A_2A_ receptor agonists have recently been shown to mitigate FT burn wound healing by dampening the inflammatory response in a porcine burn wound model [61]. Adenosine concentrations have also been reported to be elevated in human burn wound blister fluids [62].

In their simplest interpretation, metabolite abundances reflect changes to organic matter within the wound that may originate from altered cell infiltration, the extracellular matrix, angiogenesis, or the grafting material. In addition to adenosine, most metabolites identified were increased at day 7 in AFSG compared to FBD in PT wounds. It can, however, not be ruled out that changes in the metabolite abundance simply reflect a different organic load introduced into the wound by the different grafting materials. AFSGs and FBD are primarily protein-based, and the amino acid quantity released from the breakdown of these materials is ultimately dependent upon the amount of grafted material. However, in FT wounds as opposed to PT wounds, most metabolites were decreased in AFSG-treated burns as compared to cadaver skin-treated burns, consistent with the increased mass/thickness of cadaver skin, with the notable exception of adenosine and glutamate. In light of the well-documented effects of adenosine on wound healing outlined in the previous paragraphs and increased adenosine in wounds treated with AFSG, an emerging hypothesis from this study is that altered purinergic signaling contributes to suppressed inflammation and altered ROS, increased wound closure rates, and re-epithelialization compared to FBD. A more mechanistic approach, however, would be required to support this hypothesis.

In addition to adenosine, we also found adenine, S-adenosyl-L-homosysteine (SAH), S-adenosyl-methionine, and cystathionine to be significantly higher in the DPT wounds treated with AFSGs. These compounds are part of the methionine cycle and are connected through the catabolism of SAH to homocysteine and adenosine and the subsequent conversion of homocysteine to cystathionine (Figure 6). Given the increases in the abundances of these metabolites, we took a closer look into the concentrations of six more of the compounds that form the methionine cycle and compared them between wounds treated with the two biomaterials. As shown in Figure 6, despite the fact that the differences found were not significant for all of the metabolites within this pathway, four of the investigated compounds (methionine, S-adenosyl methionine, homocysteine, and GSH) were higher in abundance in the AFSG treated wounds. These results suggest that the methionine cycle is altered in the early stage of healing when burn wounds are treated with AFSGs. In cells, the methionine cycle is the major contributor to the levels of S-adenosyl methionine, the universal methyl donor that serves as a substrate for all methylation reactions, including those that modulate gene expression (via methylation of DNA, RNA, and histones), phospholipid integrity, the activity of signaling pathways, and polyamine biosynthesis. Moreover, methionine contributes to essential metabolic pathways that regulate nucleotide biosynthesis and intracellular redox balance via one-carbon metabolism. The latter is achieved by providing homocysteine as a substrate for the trans-sulfuration pathway, which ultimately produces the antioxidant glutathione.

Although the importance of methionine in maintaining oxidative homeostasis is well documented [63], the association between the methionine cycle and wound healing has been scarcely studied, and the few existing studies have mainly investigated the effect of dietary methionine on chronic wounds [64,65]. The activity within the methionine salvage pathway is dependent upon the coenzyme vitamin B_12_ for the conversion of homocysteine to methionine through homocysteine methyltransferase. Vitamin B_12_ on its own or combined with other vitamin B derivatives has been reported to enhance keratinocyte wound closure rates in vitro [66], increase tensile strength [67] and increase diabetic wound healing in mice [68], and the deficiency of the vitamin B_12_ is associated with poor healing of diabetic foot ulcers [69]. Based upon these findings, our data indicate that the properties of AFSG wound treatment are, in part, attributed to changes to the methionine salvage pathway.

While untargeted omics is a powerful tool that can provide information on changes to thousands of variables, it is limited by the confident structural annotation of the identified *m*/*z* features. Our ability to identify only 5–10% of the thousands of features that were measured is, however, in accordance with similar studies [70,71]. Moving forward, a targeted quantitative metabolomics approach could be taken, which would allow more confident metabolite annotations and targeted coverage of metabolic pathways. In addition, our approach does not discriminate between cell intrinsic and extrinsic metabolism or cell/tissue-specific alterations. Although technically challenging in execution, such an approach, combined with assessing the impact of biomaterials on the development of wound healing processes at earlier timepoints during healing, would afford a better understanding of how biomaterials impact metabolism during healing.

## 5. Conclusions

The metabolic changes during wound healing of DPT and FT wounds follow three basic trajectory patterns during the first 60 days of healing. We identified metabolic biomarkers that may be used as indicators of burn wound healing progression in future studies. Furthermore, we reported that biomaterials differentially affect the metabolome of wounds in the early stages of healing. We identified several metabolites, including adenosine, which are known to play significant roles in the biochemical processes that underlie wound healing, along with changes to metabolites within the methionine salvage pathway whose functional significance to burn wound healing remains unexplored.

## Data Availability

Data is contained within the article and Supplementary Material.

## Supplementary Materials

The following supporting information can be downloaded at https://www.mdpi.com/article/10.3390/antiox12122076/s1. Figure S1. Identification of *m/z* features that respond to treatment at day 7 during healing. Comparison of *m/z* feature intensities detected in burn wounds treated with either AFSG or FBD xenograft. A total of 585 *m/z* features identified in positive ion mode (A) and negative ion mode (B) changed depending on the xenograft used to treat PT burn wounds. Simlarly, total of 315 *m/z* features identified in positive ion mode (C) and negative ion mode (D) changed depending on the xenograft used to treat FT burn wounds. (Students’ *t*-test, FDR Bonferroni, *p* < 0.05, FC > 1.5). Table S1: Summary of raw data and statistical analysis. Table S2: Annotations of selected *m/z* features.
