# Supplementary material for: Fish Skin Grafts Affect Adenosine and Methionine Metabolism during Burn Wound Healing
Source: Antioxidants (Basel). 2023 Dec 5;12(12):2076. doi: 10.3390/antiox12122076 (PMC10741162; doi:10.3390/antiox12122076)
Supplement: Supplementary file 1 [file antioxidants-12-02076-s001.zip › Supplementary Figure 1.pdf]

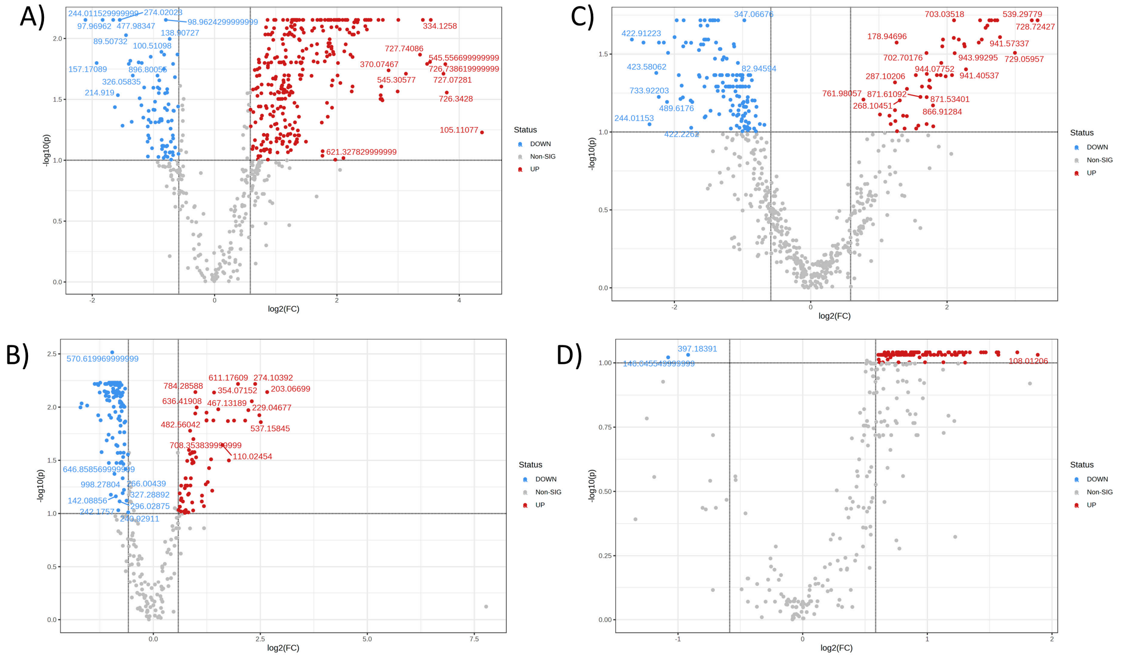

Supplementary Figure 1. Identification of m/z features that respond to treatment at day 7 during healing. Comparison of m/z feature intensities detected in burn wounds treated with either AFSG or FBD xenograft. A total of 585 m/z features identified in positive ion mode (A) and negative ion mode (B) changed depending on the xenograft used to treat PT burn wounds. Simlarly, total of 315 m/z features identified in positive ion mode (C) and negative ion mode (D) changed depending on the xenograft used to treat FT burn wounds. (Students's t-test, FDR Bonferroni, *P*<0.05, FC>1.5)
